# A Real-Time PCR Assay for the Diagnosis of Intestinal Schistosomiasis and Cure Assessment After the Treatment of Individuals With Low Parasite Burden

**DOI:** 10.3389/fimmu.2020.620417

**Published:** 2021-03-16

**Authors:** Liliane Maria Vidal Siqueira, Carolina Senra, Áureo Almeida de Oliveira, Nidia Francisca de Figueiredo Carneiro, Luciana Inácia Gomes, Ana Rabello, Paulo Marcos Zech Coelho, Edward Oliveira

**Affiliations:** ^1^ Diagnosis and Therapy of Infectious and Oncologic Diseases, Instituto René Rachou, Oswaldo Cruz Foundation, Belo Horizonte, Brazil; ^2^ Clinical Research and Public Politics in Infectious and Parasitic Diseases, Instituto René Rachou, Oswaldo Cruz Foundation, Belo Horizonte, Brazil; ^3^ School of Medicine, Universidade Estadual de Montes Claros (UNIMONTES), Montes Claros, Brazil

**Keywords:** intestinal schistosomiasis, laboratory diagnosis, Kato–Katz technique, saline gradient technique, real-time PCR

## Abstract

The laboratorial diagnosis of the intestinal schistosomiasis is always performed using Kato-Katz technique. However, this technique presents low sensitivity for diagnosis of individuals with low parasite burden, which constitutes the majority in low endemicity Brazilian locations for the disease. The objective of this study was developed and to validate a real-time PCR assay (qPCR) targeting 121 bp sequence to detect *Schistosoma* spp. DNA for the diagnosis of intestinal schistosomiasis and a sequence of the human β-actin gene as internal control. Firstly, the qPCR was standardized and next it was evaluated for diagnosis and cure assessment of intestinal schistosomiasis in the resident individuals in Tabuas and Estreito de Miralta, two locations in Brazil endemic for intestinal schistosomiasis. The qPCR assay results were compared with those of the Kato-Katz (KK) test, examining 2 or 24 slides, Saline Gradient (SG) and “reference test” (24 KK slides + SG). The cure assessment was measured by these diagnostic techniques at 30, 90, and 180 days post-treatment. In Tabuas, the positivity rates obtained by the qPCR was 30.4% (45/148) and by “reference test” was of 31.0% (46/148), with no statistical difference (p = 0.91). The presumed cure rates at 30, 90, and 180 days post-treatment were 100, 94.4, and 78.4% by the analysis of 24 KK slides, 100, 94.4, and 78.4% by the SG, and 100, 83.3, and 62.1% by the qPCR assay. In Estreito de Miralta, the positivity obtained by qPCR was 18.3% (26/142) and with “reference test” was 24.6% (35/142), with no statistical difference (p = 0.20). The presumed cure rates were 93.3, 96.9, and 96.5% by the KK, 93.3, 96.9, and 100% by the SG, and 93.3, 93.9, and 96.5% by the qPCR at 30, 90, and 180 days post-treatment, respectively. This study showed that the diagnostic techniques presented different performance in the populations from the two districts (Tabuas and Estreito de Miralta) and reinforces the need of combining techniques to improve diagnosis accuracy, increasing the detection of individuals with low parasite burden. This combination of techniques consists an important strategy for controlling the disease transmission.

## Introduction

In Brazil, intestinal schistosomiasis is caused by *Schistosoma mansoni*, the only species with established transmission. Despite the prevalence and parasite burden having decreased over the years after implanting the preventive measurements of the Schistosomiasis Control Program, in 1975, the disease still occurs in Brazil. Intestinal schistosomiasis is currently found in low, moderate, and high endemicity areas of 19 Brazilian federal units (1). The last national prevalence survey (INPEG 2010–2015) estimated 1,500,000 positive individuals for intestinal schistosomiasis in Brazil, which remains an important public health issue (2).

This situation could be partly attributed to the lack of accurate diagnostic techniques to detect intestinal schistosomiasis in endemic areas. The use of the Kato-Katz technique to detect *S. mansoni* eggs (3) with one or two slides from a single fecal sample per individual is extensively employed in prevalence surveys and individual diagnosis due to its practicability and low cost (4). This technique is sensitive to the diagnosis of *S. mansoni* infection when applied in fecal samples from individuals with moderate and high parasite burden. However, the lack of sensitivity presented by this technique occurs when it is used to diagnose individuals with low parasite burden, who are mostly present in low endemicity area (5–11). The *S. mansoni* infected individuals who were not diagnosed contribute to maintain the local transmission or to establish new outbreak when they migrate to a non-endemic area, hindering the efficacy of the control measures.

Serological assays have been used for schistosomiasis diagnosis by detecting antibodies against schistosomal antigens. However, they are unable to discriminate between active infections and past exposures, especially in individuals living in regions endemic for schistosomiasis (12). A lateral flow cassette assay was developed to overcome the limitation of parasitological and serological techniques to detect circulating cathodic antigen in urine from the *Schistosoma* infected individuals (POC-CCA^®^, Rapid Medical Diagnostics, Pretoria, South Africa). This test became available in 2003 and seems to be more sensitive than the Kato-Katz technique when applied in areas highly endemic for *S. mansoni* (13). However, there are controversies regarding the sensitivity of the POC-CCA when applied in individuals from low endemicity areas. In these cases, the POC-CCA has showed larger sensitivity only in patients with moderate or high parasite burden (14, 15).

Alternatively, the detection of schistosome DNA through DNA amplification techniques provides advantages compared to the many parasitological techniques and serological tests, due their high sensitivity, specificity, and accuracy. Furthermore, DNA amplification techniques can detect early pre-patent infections. Although the PCR assay is widely used for laboratory diagnosis of many infectious and parasitic diseases, its application for schistosomiasis was reported for the first time for our research group. We showed that PCR targeting 121 bp, described by Hamburger et al. (16) achieved a limit of detection (LOD) of 1 fg of *S. mansoni* egg template DNA and absence of amplification of the DNA from *Ascaris lumbricoides*, *Ancylostoma duodenale*, *Taenia solium*, and *Trichiuris trichiuria*, helminths commonly found in the same endemic areas.

Since then, we have extensively worked with this 121 bp sequence as a target in the PCR assays for diagnosing intestinal schistosomiasis. The 121 bp sequence was used successfully in conventional PCR (17, 18), PCR-ELISA (19, 20), obtaining consistent results. Furthermore, other studies show the 121 bp sequence as a target in a real-time PCR and oligochromatography-polymerase chain reaction with higher sensitivity than the Kato-Katz technique for diagnosing intestinal schistosomiasis (21, 22). Moreover, the 121 bp DNA sequence was targeted to detect *Schistosoma* DNA in plasma (23) and urine samples (24, 25) using conventional PCR.

Thus, the main goal of this study was to develop a qPCR assay targeting 121 bp sequence to detect *S. mansoni* DNA in fecal samples to diagnose intestinal schistosomiasis and assess the post-treatment cure for individuals with low parasite burden. In addition, a 92 bp sequence from the human β-actin gene too was amplified in the same reaction as internal control for ensure the efficiency of DNA extraction and PCR-amplification.

## Material and Methods

### qPCR Assay Standardization

#### Extraction of *S. mansoni* DNA

In this study we tried contaminate negative fecal samples with *S. mansoni* eggs and we did not have success. The *S. mansoni* eggs are relatively big ones and we had difficulties to count the *S. mansoni* eggs in Neubauer chamber and then recover it to contaminate negative fecal samples.

To contorn this limitation, genomic DNA was extracted from adult *S. mansoni* worms (BH strain) obtained from the liver of Swiss albino mice 60 days after infection with 150 cercariae using QlAamp DNA Mini and Blood Mini Handbook (QIAGEN, GmbH, Hilden, Germany), following the manufacturer`s protocol.

As negative controls we used DNA extracted from three negative *S. mansoni* fecal samples collected from children resident in non-endemic area who had negative results by Kato-Katz technique. The total DNA was extracted using the QIAamp DNA Stools Mini Kit (Qiagen GmbH, Hilden, Germany), according to the manufacturer’s recommendations and following the protocols of DNA Isolation from Stool for Pathogen Detection and DNA Isolation from Large Amounts of Stool. The DNA concentration and A260/A280 absorbance ratio was measured in a Nanodrop ND-1000 spectrophotometer (Thermo Fisher Scientific, Wilmington, DE, USA) to ensure the efficiency of the DNA extraction and to verify the purity of the DNA obtained.

### Primers and Probes

**Figure 1:**
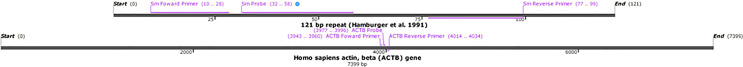
Diagram showing anneling positions of the primers and probes in the 121 bp and human β-actin gene sequences. SnapGene software (from Insightful Science; available at snapgene.com).

A forward 5′-CCG ACC AAC CGT TCT ATG A-3′ and reverse 5′-CAC GCT CTC GCA AAT AAT CTA AA-3′ primers and a 5′-6[FAM]/TCG TTG TAT CTC CGA AACCAC TGG ACG/[3BHQ1] probe were designed to amplify and detect a 90 bp fragment of a highly repetitive 121 bp sequence of *S. mansoni* (GenBank: M61098). A forward 5’-CCA TCT ACG AGG GGT ATG-3’ and reverse 3’-GGT GAG GAT CTT CAT GAG GTA-5’ primers, and the 56-JOE/CCT GCG TCT GGA CCT GGC TG/[3BHQ1] probe were designed to amplify and detect a 92 pb of the human β-actin gene (GenBank: AY582799.1) as internal control (**Figure 1**). All primers and probes were designed in the Primer3-web program 0.4.0 (26) and submitted to homology searches on the National Center for Biotechnology Information website with nucleotide BLAST program using the Nucleotide collection and Megablast option database. The primers and probes were purchased from Integrated DNA Technologies Inc. (Coralville, IO, USA). Initially, we tried *S. mansoni* primers at 0.1, 0.2, 0.3 μM and *S. mansoni* probe at 0.1, 0.25, and 0.5 μM in different combinations in the simplex qPCR assay using 38 ng, 3.8 ng, 380 pg, 38 pg, 3.8 pg, 380 fg, 0.38 fg, and 0.038 fg genomic DNA of *S. mansoni* diluted 1:5 in linear acrylamide solution [30 μg/ml (w/v) in DEPC treat H2O]. Next, we tried the human β-actin gene primers at 0.1, 0.15, and 0.2 μM and human β-actin gene probe at 0.1, 0.25, and 0.5 μM in a simplex qPCR assay using DNA extracted from negative *S. mansoni* fecal samples diluted 1:5 in linear acrylamide solution. In this way, the best qPCR protocol was defined as:

The reaction was performed with a final volume of 25 μl containing 12.5 μl of TaqMan^®^ Universal PCR Master Mix (Life Technologies, Thermo Fisher Scientific Inc., USA), *S. mansoni* specific primers at 0.1 μM, 5′-6[FAM]—[3BHQ1] probe at 0.25 μM, β-actin specific primers at 0.15 μM and 56-JOE—[3BHQ1] probe at 0.25 μM, BSA 0.01 μg/μl, MgCl_2_ at 2 μM and 4 μl of DNA diluted 1:5 in linear acrylamide solution. Two controls were used for each reaction, a positive control (PCR mix plus DNA extracted from adult worms) and a negative control consisting of PCR mix (No Template Control). The assays were performed in duplicate using microplates (MicroAmp^®^ Fast Optical/Applied Biosystems Foster City, CA, USA) sealed with adhesive film (Optical Adhesive Covers/Applied Biosystems) on the StepOnePlus™ Real-Time PCR System (Thermo Fisher Scientific Inc., USA) under the universal cycling program with 45 cycles and annealing temperature of 60 °C. Based on a standard curve produced with serial dilutions of *S. mansoni* DNA, samples presenting Ct ⩽ 42 were classified as positives. Samples that did not presented internal control JOE (β-actin Probe) amplification were retested and a new DNA sample was reextracted when necessary.

Extraction and amplification protocols were performed in different rooms to minimize the possibility of contamination. All experiments were performed in a laminar flow chamber, previously irradiated with ultraviolet light, and employing only sterile disposable products, including barrier tips.

### Analytical Sensitivity (Limit of Detection)

The lower LOD of the qPCR was defined by the amplification curve of a positive control containing 38 ng, 3.8 ng, 380 pg, 38 pg, 3.8 pg, 380 fg, 0.38 fg, and 0.038 fg of genomic DNA of adult worms diluted 1:5 in linear acrylamide solution, in triplicate. The mean of Ct from the triplicates was used to define the point in the amplification curve. The amplification efficiency assay was analyzed according to the amplification efficiency (*E*), Slope, and R^2^, following recommendations of Johnson et al. (27).

### Analytical Specificity

DNA from *Ancylostoma duodenale*, *Ascaris lumbricoides*, and *Fasciola hepatica*, ceded by professors from the Department of Parasitology, Biology Institute, of the Universidade Federal de Minas Gerais, was used in the qPCR assay to evaluate the analytical specificity. *A. duodenale* and *A. lumbricoides* are frequently found in *S. mansoni* co-infections and *F. hepatica* is a worm phylogenetically next to the *S. mansoni*.

### Precision Tests

The repeatability test was carried out using six DNA samples extracted from human feces (three negatives and three positives for the presence of *S. mansoni* eggs), according to the Kato-Katz technique. The repeatability test was measured by the coefficient of variation (CV) by retesting four times the same samples in a single assay (intra-assay test). The reproducibility test was measured by the coefficient of variation (CV) of retesting positive control containing 38 ng, 3.8 ng, 380 pg, 38 pg, 3.8 pg, 380 fg and 0.38 fg of genomic DNA of adult worms diluted 1:5 in linear acrylamide solution in three different days.

### qPCR Validation

**Figure 2:**
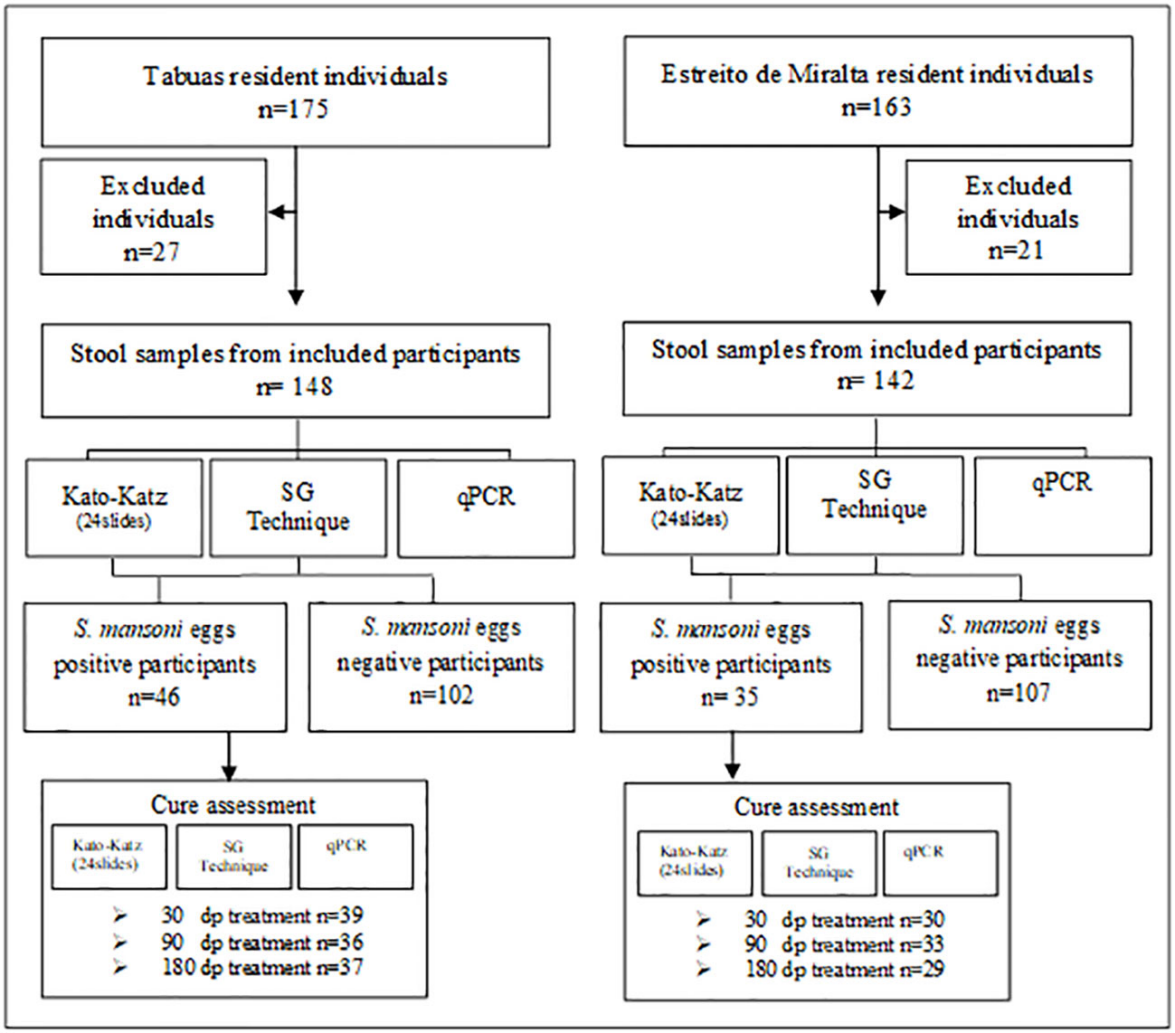
STARD flow diagram followed to select the studied groups.

The validation of the qPCR was performed through a cross-sectional-based study carried out in the Tabuas and Estreito de Miralta districts (**Figure 2**), two communities endemic for schistosomiasis from the rural area of ​​the municipality of Montes Claros, in the northern region of the state of Minas Gerais, Brazil. The prevalence in Tabuas in 2010 was estimated in 29.1% using two slides in the Kato-Katz technique in the Zoonozis Control Center of Montes Claros. There were no prevalence data from the Estreito de Miralta. However, this community was close to Tabuas and no control has been placed in these communities in the 2 years prior to the current study.

All residents of the mentioned locations with over 1 year of age, of both gender, and who agreed to participate in the study and signed the informed consent form were included. Furthermore, the diagnostic tests were applied to assess the cure after specific treatment of low parasite burden individuals.

### Stool Samples

The stools samples were provided by the participants at day 0 and examined using the Kato-Katz and Saline Gradient techniques in both locations. The participants who presented *S. mansoni* eggs in their stools were treated with 60 mg/kg of praziquantel for children and 50 mg/kg for adults. New stools samples were collected at 30, 90, and 180 days post-treatment, totalizing four samples by participant until the study end. The participants who presented eggs or cyst of other parasites were treated with 400 mg of albendazole (single oral dose) as recommended by the Brazilian Ministry of Health.

### Kato–Katz Technique

The fecal samples from the residents of Tabuas and Estreito de Miralta were submitted to the Kato-Katz technique using the Helm-Test^®^ produced by BioManguinhos-Fiocruz (Rio de Janeiro, RJ, Brazil). Twenty-four slides of the same stools sample, which correspond to the 1,000 mg of feces, were examined to perform a quantitative comparison between the parasitological tests. Infection intensity was calculated by the number of *S. mansoni* eggs found in 24 slides, resulting in eggs per gram of feces (epg). According to WHO (4), the *S. mansoni* infection intensity is classified as light (1–100 epg), moderate (101–400 epg), and high (>400).

### Saline Gradient Technique

The Saline Gradient technique was performed according to the protocol published by Coelho et al. (28). Fecal samples were filtered through nylon screen (150 µm) and two portions of 500 mg were quantified using a metal plate. The portions were subjected to a slow flow of a 3% saline solution for 1 h. Subsequently, the system was closed and all remaining material transferred to a Falcon^®^ tube (15 ml), after which 20% formaldehyde was added to the sediment obtained (approximately 2 ml of sediment). The final solution was examined in an optical microscope. All sediment was examined, the helminth eggs were counted, and the *S. mansoni* eggs were separated in two preparations (500 mg + 500 mg) representing eggs per gram of feces (epg).

### qPCR Assay

The DNA of 1,000 mg fecal samples obtained from residents of Tabuas and Estreito de Miralta was extracted using the QIAamp DNA Stools Mini Kit (Qiagen GmbH, Hilden, Germany), according to the manufacturer’s recommendations and following the protocols of DNA Isolation from Stool for Pathogen Detection and DNA Isolation from Large Amounts of Stool. The DNA concentration was measured by absorbance at 260 nm in a Nanodrop ND-1000 spectrophotometer (Thermo Fisher Scientific, Wilmington, DE, USA). The A260/A280 absorbance ratio was analyzed to verify the purity of the DNA obtained.

The qPCR assay was performed using fecal samples according to the conditions standardized. The DNA samples that did not present amplification for the human β-actin gene were retested for ensure the efficiency of DNA extraction and PCR-amplification.

### Data Analysis

The database was built in Microsoft Office Excel 2007 spreadsheets and analyzed using GraphPad Prism version 6.0 (San Diego, CA, USA) or Open Epi software version 3.0 (29). The positivity, sensitivity, specificity, and accuracy rates of the parasitological and molecular tests were calculated using the Open Epi software. The chi-square test was used for comparisons between proportions considering a 5% significance level (30). The degree of agreement between diagnostic tests was determined by the Kappa index and interpreted according to Landis & Koch (31). Correlations between epg from the KK and Ct from the qPCR test results were tested using the Spearman’s coefficiency of correlation.

### Ethical Approval

The use of human samples was approved following the standards of the Ethical Review Committee of the IRR/FIOCRUZ, Brazil (CEPSH 03/2008) and National Committee of Ethical Research (784/2008, CONEP 14886) in accordance with the Brazilian legislation (RDC 466/2012). The written informed consent was obtained from all the participants/parents or guardians before collecting the samples.

## Results

### qPCR Assay Standardization

A standard curve was constructed and the analytical sensitivity assay showed that the *S. mansoni* DNA was detected up to the seventh dilution, which corresponds to 0.38 fg. Moreover, repeatability and linearity in the standard curve were obtained up to Ct 41 (CV ranging from 0.05 to 2.7%; Slope: −3.222; *E*: 104%, and R²: 0.98).

The analytical specificity was assessed using DNA from *Ancylostoma duodenale*, *Ascaris lumbricoides*, and *Fasciola hepática* adult worms. The results showed no unspecific amplifications when using genomic DNA in the qPCR assay. The repeatability test presented acceptable Ct variations in the four replicates of three *S. mansoni* negative and three *S. mansoni* positive samples. In this assay the coefficient of variation (CV) were 1.74, 2.18, and 2.33% for the target (FAM probe) and 0.69, 0.48, and 0.40% for internal control (JOE probe). Likewise, the reproducibility test presented Ct consistent, resulting CV ranging from 1.6 to 5.4%.

## qPCR Validation in Tabuas

### Positivity Rates for *S. mansoni* and Other Parasites

In Tabuas, 84.5% (148/175) of the population participated of this study. From these, 73 were females and 75 males, aged between 1 and 86 years. Ninety-six individuals were residents in the Tabuas district and 52 in the Ribeirão de Tabuas, an adjacent location. The reasons for non-participation in the study were: 1) refusal; 2) insufficient biological sample for performing all techniques, and 3) health reasons.

**Figure 3:**
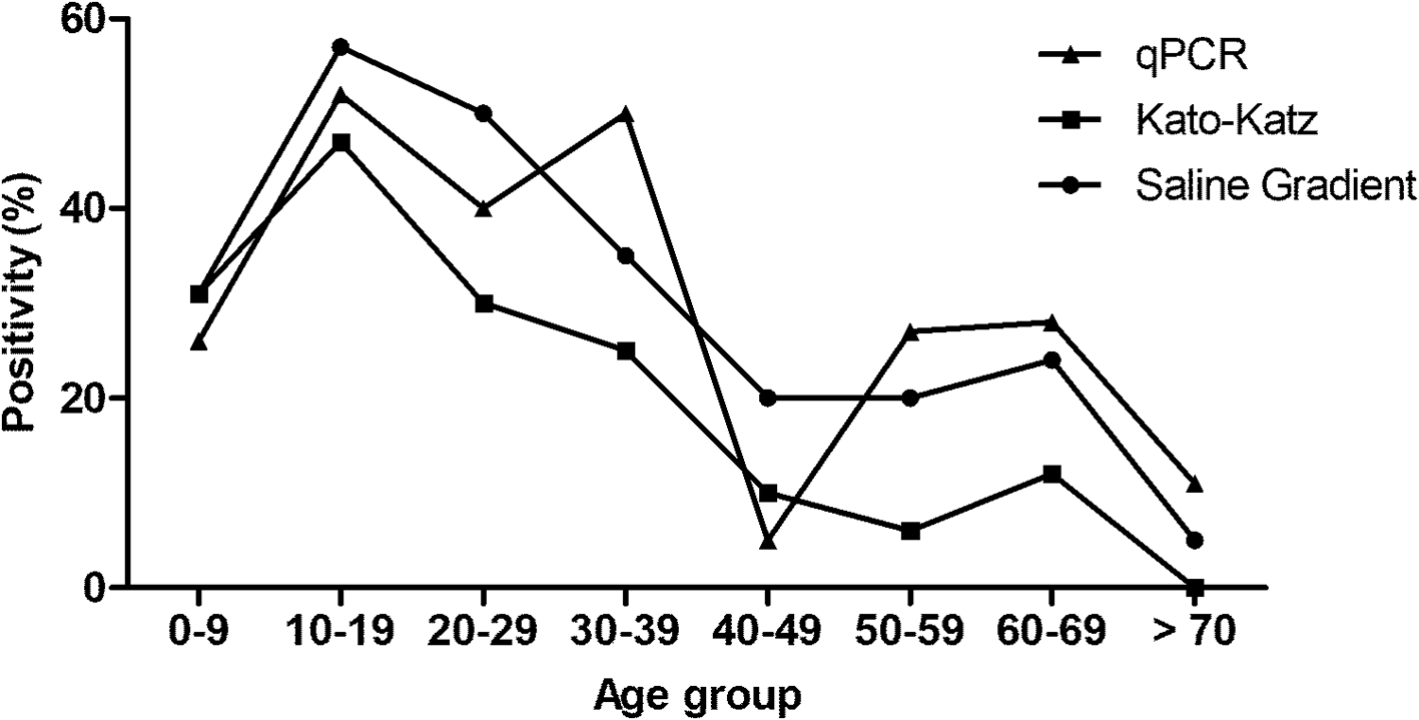
Positivity of intestinal schistosomiasis in the participants from Tabuas district, diagnosed by Kato-Katz (24 slides), Saline Gradient, and qPCR and distributed by age ranges.

The **Table 1** shows the positivity rates found in the population from Tabuas. The positivity rate of the KK technique was 12.1% (18/148), 15.5% (23/148), 16.9% (25/148), 19.6% (29/148), 19.6% (29/148), and 20.6% (31/148) for 1, 2, 3, 6, 12, and 24 slides examined, respectively. The higher positivity rates were found in individuals with 10 to 19 (47.6%) and 20–29 years of age (30%) (**Figure 3**). Of the 31 positive participants, 25 (80.7%) presented low parasite load (1–100), five (16.1%) presented moderate load (101–400), and one (3.2%) presented high parasite load (>400 epg).

**Table 1:** Positivity rates of intestinal schistosomiasis found by parasitological techniques and qPCR in the population from Tabuas district, Minas Gerais state, Brazil.

	Kato–Katz (n = 148)						Saline gradient (n = 148)	“Reference test” (n = 148)	qPCR (n = 148)
	One slide	Two slides	Three slides	Six slides	Twelve slides	Twenty-four slides	1,000 mg of feces	KK (24 slides) plus SG Results	1,000 mg of feces
Positivity (%)	12.2 (7.2–19.2)^*^	15.5 (9.9–23.3)	16.9 (10.9–24.9)	19.6 (13.1–28.1)	19.6 (13.1–28.1)	20.6 (14.2–29.7)	29 (21–39.1)	31 (22.8–41.5)	30.4 (22.2–40.7)

^*^95% Confidence interval.

The SG technique detected 43/148 participants with *S. mansoni* eggs in their stools, producing a positivity rate of 29.0%. Likewise, the SG technique showed higher positivity rates in the age range from 10 to 19 (57.1) followed by 20–29 years (50%) (**Figure 3**). Of the 43 positive participants, 40 had low parasite load and only three presented moderate loads.

To create a “reference test,” the results obtained by KK (24 slides) and SG were combined and the positivity increased to 31.0% (46/148), with a statistical difference regarding the previous KK positivity (*p* = 0.04), although without statistical difference compared to SG (*p* = 0.70). The positivity rate obtained by the qPCR assay was 30.4%, represented by 45/148 positive participants. All samples (148/148) presented amplification of the internal control. The highest positivity rates for this assay occurred in the age ranges from 10 to 19 (52%) and 30 to 39 years (50%) (**Figure 3**).

Besides *S. mansoni*, the two combined techniques detected 37 positive participants for other parasites, 16 (10.8%) positive for hookworms, eight (5.4%) for *Enterobius vermicularis*, eight (5.4%) for *Ascaris lumbricoides*, six (4%) for *Giardia* sp., 20 (13.5%) for *Entamoeba coli*, two (1.4%) for *Trichuris trichiura*, one (0.68%) for *Taenia* sp., and two (1.4%) for *Hymenolepis nana*. Of the participants infected with *S. mansoni*, six presented co-infection with hookworms and *E. vermicularis*.

### qPCR Performance in Different Scenarios

Considering the results of the KK technique with two slides as a definitive diagnostic, the qPCR presented 95.7% sensitivity (95% CI: 79–99.2), 81.6% specificity (95% CI: 73.9–87.4), and 83.8% accuracy (95% CI: 77–88.9). Considering the results of the KK technique with 24 slides, the qPCR presented 96.7% sensitivity (95% CI: 83.8–99.4), 87.2% specificity (95% CI: 79.9–92.0), and 89.2% accuracy (95% CI: 83.2–93.2).

Considering the SG results, the qPCR presented 81.4% sensitivity (95% CI: 67.3–90.2), 90.5% specificity (95% CI: 83.3–94.7), and 87.8% accuracy (95% CI: 81.6–92.2). Based in the results from the “reference test,” the qPCR presented 82.6% (95% CI: 69.3–90.9), 93.1% (95% CI: 86.5–96.6), and 89.8% (95% CI: 83.9–93.7) of sensitivity, specificity, and accuracy rates, respectively (**Table 2**).

**Table 2:** Performance of qPCR considering the parasitological techniques and “reference test” applied in the population from Tabuas district, Minas Gerais state, Brazil.

	Kato-Katz (2 slides) as reference (n = 148)			Kato-Katz (24 slides) as reference (n = 148)			Saline gradient as reference (n = 148)			“Reference test” as reference (n = 148)		
	Sensitivity (%)	Specificity (%)	Accuracy (%)	Sensitivity (%)	Specificity (%)	Accuracy (%)	Sensitivity (%)	Specificity (%)	Accuracy (%)	Sensitivity (%)	Specificity (%)	Accuracy (%)
qPCR	95.7 (79–99.2)*	81.6 (73.9–87.4)	83.8 (77–88.9)	96.8 (83.8–99.4)	87.2 (79.9–92.4)	89.2 (83.2–93.2)	81.4 (67.4–90.3)	90.5 (83.4–94.7)	87.8 (81.6–92.2)	82.6 (69.3–90.9)	93.1 (86.5–96.6)	89.9 (84–93.8)

^*^95% Confidence interval.

**Table 3** shows the agreement ratios between the parasitological techniques and qPCR results. Among the 45 participants positive to *S. mansoni* by the qPCR assay, 22 were consistent with the KK technique (two slides), one presented positive KK and negative qPCR results (with positive amplification of human β-actin gene), and 23 were positive only by the qPCR (Kappa index: 0.56). On the other hand, 30 participants were consistent with the KK technique (24 slides), one presented positive KK and negative qPCR results (with positive amplification of human β-actin gene), and 15 were positive only by qPCR (Kappa índice: 0.72). Furthermore, there is a negative correlation between the microscopic egg counts (epg) and Ct (r: −0.404) obtained by the KK technique (24 slides) and qPCR assay, respectively.

**Table 3:** Agreement analysis of the qPCR results in relation to the parasitological techniques and reference test in the population from Tabuas district, Minas Gerais state, Brazil.

		Kato–Katz (2 slides)			Kato-Katz (24 slides)			Saline gradient			Reference test		
		P	N	T	P	N	T	P	N	T	P	N	T
qPCR	P	22	23	45	30	15	45	35	10	45	38	7	45
	N	1	102	103	1	102	103	8	95	103	8	95	103
	T	23	125	148	31	117	148	43	105	148	46	102	148
Kappa index		0.56 (0.41–0.7)			0.72 (0.56–0.88)			0.71 (0.55–0.87)			0.76 (0.6–0.92)		

P, Positive; N, Negative; T, Total; ( ), Confidence interval with 95%.

In the crosstabulation between SG and qPCR, eight participants presented positive results with the SG technique and negative with the qPCR (with positive amplification of human β-actin gene). On the other hand, 10 participants presented negative results with the SG technique but were positive with the qPCR. Thirty-five positive and 95 negative results were consistent between the SG technique and qPCR assay (Kappa index: 0.71). Forty-five individuals were positive by the qPCR, of which seven cases were not detected by the “reference test.” In contrast, eight cases were positive by the “reference test” and were not detected by the qPCR (with positive amplification of human β-actin gene), resulting in a Kappa index of 0.76.

### Cure Assessment in Tabuas

**Table 4** shows the follow-up for cure assessment. Of the 46 positive participants treated with praziquantel, 39 participated of the follow-up for cure assessment at 30 days post-treatment. Of these individuals, all presented negative results by the parasitological techniques and qPCR assay, resulting in a presumed cure rate of 100%. At 90 days post-treatment, 36 participants were reexamined for cure assessment. Among these, three participants were positive for *S. mansoni* eggs, one of which was diagnosed by both techniques (KK and SG) and two identified separately by each parasitological technique. qPCR diagnosed six positive participants at 90 days. Therefore, the presumed cure rates at 90 days post-treatment were 94.4% for the KK and SG techniques and 83.3% for the qPCR assay. At 180 days post-treatment, new stool samples were collected from 37 participants for cure assessment, of which eight were positive for *S. mansoni* eggs by both parasitological techniques and 14 were positive for *S. mansoni* DNA by the qPCR. The presumed cure rates were 78.4% for the KK and SG techniques and 62.1% for the qPCR assay.

**Table 4:** Presumed cure rates measured by parasitological techniques and qPCR 30, 90, and 180 days pos treatment of the *S. mansoni* positive participants from Tabuas district, Minas Gerais state, Brazil.

Diagnostic tests	Assessment at the 30 days	Assessment at the 90 days	Assessment at the 180 days
Kato-Katz (24 slides)	100% (39/39)^*^	94.4% (34/36)	78.4% (29/37)
Saline gradient	100% (39/39)	94.4.2% (34/36)	78.4% (29/37)
qPCR	100% (39/39)	83.3% (30/36)	62.1% (23/37)

^*^Relation of treated and presumably cured participants in each assessment moment pos treatment.

Kato-Katz (24 slides) and Saline gradient: difference between 30 and 90, p = 0.14; 90 and 180 days post treatment, p = 0.05.

qPCR: difference between 30 and 90, p = 0.008; 90 and 180 days post treatment, p = 0.04.

### qPCR Validation in Estreito de Miralta Positivity Rates for *S. mansoni* and Other Parasites

In Estreito de Miralta, 87.1% (142/163) of the population was included in this study, of which 77 were females and 65 males, aged from 01 to 86 years, 96 residents of the Estreito de Miralta and 46 of Serra Verde, an adjacent location. The reasons for non-participation in the study were the same as those considered for the Tabuas location.

**Figure 4:**
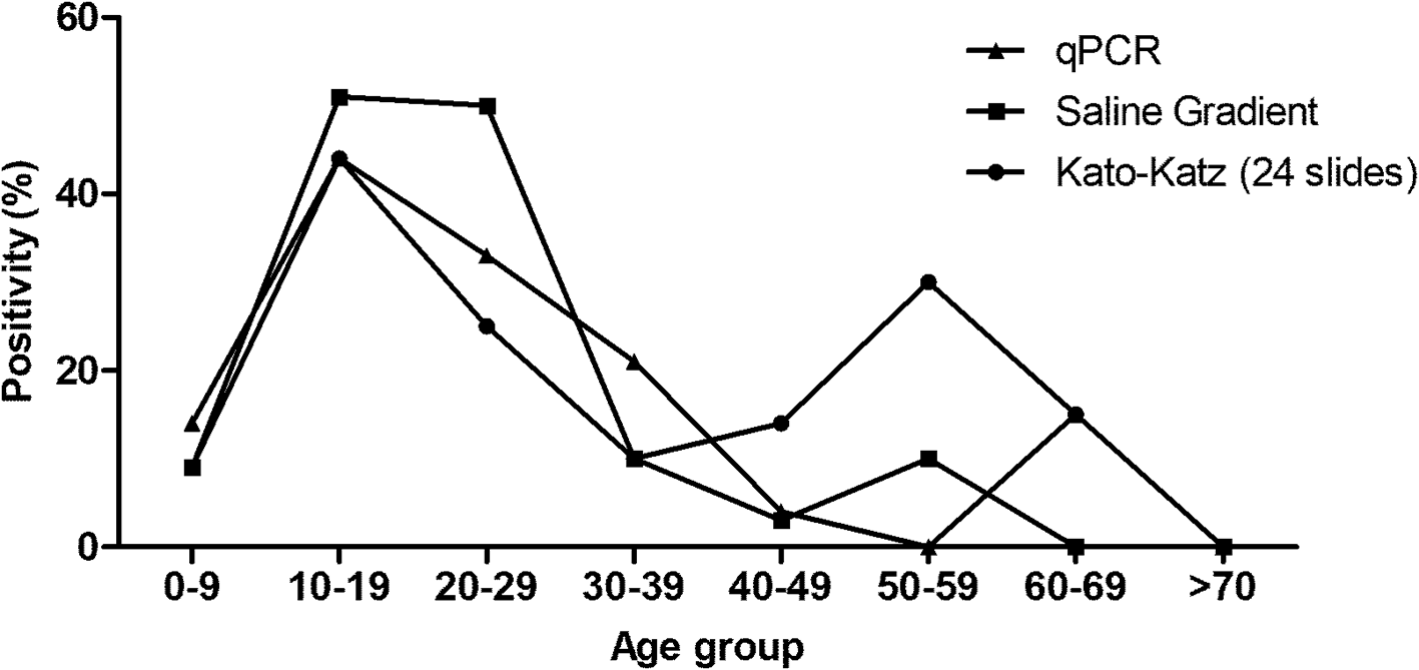
Positivity of intestinal schistosomiasis in the participants from Estreito de Miralta district, diagnosed by Kato–Katz (24 slides), Saline Gradient, and qPCR and distributed by the age ranges.

The positivity rates obtained by the KK technique were 9.2% (13/142), 10.5% (15/142), 11.3% (16/142), 12% (17/142), 16.2% (23/142), and 19.7% (28/142) using 1, 2, 3, 6, 12, and 24 slides, respectively (**Table 5**). The higher positivity rates of the KK (24 slides) technique occurred from 10 to 19 years (44.4%) and 50 to 59 years (30%) (**Figure 4**). All positive participants presented low parasitic load (1–100 epg). The SG technique detected 26/142 participants positive for *S. mansoni* eggs, with a positivity rate of 18.3% concentrated at ages from 10 to 19 (51.9%) and 20 to 29 years (50%) (**Figure 4**).

**Table 5:** Positivity rates of intestinal schistosomiasis found by parasitological techniques and qPCR in the population from Estreito de Miralta district, Minas Gerais state, Brazil.

	Kato-Katz (n = 142)						Saline gradient (n = 142)	“Reference test” (n = 142)	qPCR (n = 142)
	One slide	Two slides	Three slides	Six slides	Twelve slides	Twenty-four slides	1,000 mg of feces	KK (24 slides) plus SG Results	1,000 mg of feces
Positivity (%)	9.2 (4.9–15.7)^*^	10.5 (5.9–17.4)	11.3 (6.4–18.3)	12 (7–19.2)	16.2 (10.3–24.3)	19.7 (13.1–28.5)	18.3 (12–26.8)	24.6 (17.2–34.3)	18.3 (12–26.8)

^*^95% Confidence interval.

Likewise, “reference test” was created with the results obtained by the KK (24 slides) and SG techniques. The positivity increased to 24.6% (35/142), with no statistical difference regarding the previous KK (*p* = 0.32) and SG (*p* = 0.20) positivity rates. The positivity rate obtained by the qPCR assay was equal to the one obtained with the SG technique (18.3%), represented by 26/142 positive participants. The higher positivity rates with qPCR occurred in the age ranges from 10 to 19 years (44.4%) (**Figure 4**).

Other intestinal parasites were also detected by both parasitological techniques. The KK and SG techniques detected 14 (9.9%) individuals positive for hookworms, eight (5.6%) for *Enterobius vermicularis*, one (0.7%) for *Ascaris lumbricoides*, and three (2.1%) for *Hymenolepis nana*. Five participants presented co-infection with *S. mansoni* and hookworms and two with *S. mansoni* and *E. vermicularis*.

### qPCR Performance in Different Scenarios

**Table 6** shows the sensitivity, specificity, and accuracy rates. Considering the results of the KK technique with two slides as a true diagnosis, the qPCR showed 80% sensitivity (95% CI: 54.8–93), 89% specificity (95% CI: 82.3–93.3), and 88% accuracy (95% CI: 81.7–92.4). On the other hand, considering the results of the KK technique with 24 slides as a true diagnosis, the qPCR assay presented 64.3% sensitivity (95% CI: 45.8–79.3), 92.9% specificity (95% CI: 86.7–96.4), and 87.3% accuracy (95% CI: 80.9–91.8). Considering the SG results as reference, the qPCR presented 69.2% sensitivity (95% CI: 50–83.5), 93.1% specificity (95% CI: 87–96.5), and 88.7% accuracy (95% CI: 82.5–92.9). However, if the “reference test” results are considered as a true diagnosis, the qPCR presented 57.1% sensitivity (95% CI: 40.8–72), 94.4% specificity (95% CI: 88.3–97.4), and 85.2% accuracy (95% CI: 78.4–90.1) (**Table 6**).

**Table 6:** Performance of qPCR considering the parasitological techniques and “reference test” applied in the population from Estreito de Miralta district, Minas Gerais state, Brazil.

	Kato–Katz (2 slides) as reference (n = 142)			Kato–Katz (24 slides) as reference (n = 142)			Saline gradient as reference (n = 142)			“Reference test” as reference (n = 142)		
	Sensitivity (%)	Specificity (%)	Accuracy (%)	Sensitivity (%)	Specificity (%)	Accuracy (%)	Sensitivity (%)	Specificity (%)	Accuracy (%)	Sensitivity (%)	Specificity (%)	Accuracy (%)
qPCR	80 (54.8–93)	89 (82.3–93.3)	88 (81.7–92.4)	64.3 (45.8–79.3)	92.9 (86.7–96.4)	87.3 (80.9–91.8)	69.2 (50–83.5)	93.1 (87–96.5)	88.7 (82.5–92.9)	57.1 (40.8–72)	94.4 (88.3–97.4)	85.2 (78.4–90.1)

The qPCR results were crosstabulated with the parasitological techniques and the results are presented in the **Table 7**. Of the 142 participants, 12 were co-positive and 113 were co-negative with the KK technique (two slides) and qPCR assay, three were positive by the KK technique and negative by the qPCR assay (with positive amplification for the human β-actin gene), and 14 were positive only by the qPCR assay (Kappa index: 0.52). Twenty-six participants were positive for the qPCR assay, of which eight were not detected by the KK 24 slides. On other hand, there were 10 positive cases detected by the KK technique that the qPCR assay could not detect (Kappa index: 0.59). Moreover, is important highlight that the results of the Ct from the qPCR assay showed negative correlation (r: −0.427) with the microscopic egg counts (epg) obtained by KK technique (24 slides).

**Table 7:** Agreement analysis of the qPCR results in relation to the parasitological techniques and “reference test” in the population from Estreito de Miralta district, Minas Gerais state, Brazil.

		Kato-Katz (2 slides)			Kato-Katz (24 slides)			Saline gradient			Reference test		
		P	N	T	P	N	T	P	N	T	P	N	T
qPCR	P	12	14	26	18	8	26	18	8	26	20	6	26
	N	3	113	116	10	106	116	8	108	116	15	101	116
	T	15	127	142	28	114	142	26	116	142	35	107	142
Kappa index		0.52 (0.36–0.68)			0.59 (0.42–0.75)			0.62 (0.46–0.79)			0.56 (0.4–0.73)		

P, Positive; N, Negative; T, Total; ( ), Confidence interval with 95%.

The crosstabulation of the SG and qPCR results showed that 126 were consistent and 16 discordants. Among the discordant results, eight were qPCR positive and SG negative and eight were qPCR negative (with positive amplification for the human β-actin gene) and SG positive (Kappa index: 0.62). There were 21/142 qPCR and “reference test” discordant results, of which six presented positive qPCR and negative “reference test” results. In contrast, 15 individuals presented negative qPCR (with positive amplification of human β-actin gene) and positive “reference test” results, resulting in a Kappa index of 0.56 (**Table 7**).

### Cure Assessment in Estreito de Miralta

In the follow-up for cure assessment 30 days post-treatment, new stool samples were collected from 30/35 positive participants. This evaluation showed two participants diagnosed as positive for *S. mansoni* infection by the KK, SG techniques and qPCR assay, resulting in a presumed cure rate of 93.3%. In the evaluation 90 days post-treatment, of the 35 participants positive for *S. mansoni* who were treated with praziquantel, 33 were sampled to evaluate cure assessment. The KK and SG techniques detected two positive participants, one by each technique. The qPCR assay diagnosed both participants as positive for *S. mansoni* DNA. The presumed cure rates were 97% measured by the KK and SG techniques and 93.3% by the qPCR assay.

The follow-up at 180 days post-treatment consisted of 29/35 samples for new stool samples for the last cure assessment. This evaluation showed only one participant diagnosed with intestinal schistosomiasis by the KK technique and qPCR, resulting in a presumed cure rate of 96.5% (**Table 8**).

**Table 8:** Presumed cure rates measured by parasitological techniques and qPCR 30, 90, and 180 days post treatment of the *S. mansoni* positive individuals from Estreito de Miralta district, Minas Gerais state, Brazil.

Diagnostic tests	Assessment at the 30 days	Assessment at the 90 days	Assessment at the 180 days
Kato-Katz (24 slides)	93.3% (28/30)^*^	97% (32/33)	96.5% (28/29)
Saline gradient	93.3% (28/30)	97% (32/33)	100% (29/29)
qPCR	93.3% (28/30)	93.9% (31/33)	96.5% (28/29)

^*^Relation of treated and presumably cured participants in each assessment moment post treatment.

Kato-Katz (24 slides): difference between 30 and 90, p = 0.51; 90 and 180 days post treatment, p = 0.93.

Saline gradient: difference between 30 and 90, p = 0.51; 90 and 180 days post treatment, p = 0.34.

qPCR: difference between 30 and 90, p = 0.92; 90 and 180 days post treatment, p = 0.63.

## Discussion

Despite the parasitological technique presenting the best cost-benefit conditions, the assessment of more sensitive techniques is essential for an efficient diagnosis in endemic areas. Current scenarios show that molecular tests are a promising tool for diagnosis and cure assessment of intestinal schistosomiasis in individuals of low parasite burden (17–20, 32, 33). One of the advantages of the qPCR assay is its potential for high throughput, elimination of post-PCR handling, and possible quantification. Moreover, the qPCR can be multiplexed to detect other parasites in the feces using primers highly specific for each parasite of interest (33). In this study, the qPCR assay was duplexed to detect *S. mansoni* and the human *β*-actin gene to diagnose the intestinal schistosomiasis and to secure the optimal conditions of amplification, respectively.

In the conditions defined in this study, the qPCR was extremally sensitive and capable of detecting 0.38 fg of *S. mansoni* DNA, which corresponds to approximately 0.00065 times its genome, that contains ~580 fg of DNA (34). Thus, the LOD defined in this study (0.38 fg) corresponds to less than a single cell of this multi-cellular parasite. Among the PCR assays described in the literature, those targeting 121 bp have presented lower LOD, ranging from 1 to 3 fg of total DNA (18–20, 35).

The qPCR assay was highly specific for detecting *S. mansoni* DNA. The primers used in the qPCR are genus-specific and did not amplify the DNA of *Ancylostoma duodenale*, *Ascaris lumbricoides*, *Fasciola hepatica*, and *Ancylostoma duodenale.* Furthermore, there were no false-positive results in the stool samples collected from the participants infected with *Enterobius vermicularis*, *Giardia* sp., *Entamoeba coli*, *Trichuris trichiura*, *Taenia* sp., and *Hymenolepis nana.*

In Tabuas, the positivity rate presented by the KK technique increased until six slides and kept relatively constant from 6 to 24 slides examined of the same fecal sample (**Table 1**). This behavior was also observed by other authors, who emphasize that the positivity rate is directly proportional to the number of slides and fecal samples examined. Enk et al. (7) showed that the positivity rate of schistosomiasis in an experimental group of 305 participants increased from 13.8 to 19% when one and six KK slides were examined. Moreover, an increase of 20.7 to 27.2% in the prevalence of schistosomiasis occurred when these authors examined three stool samples. Likewise, Siqueira et al. (8) found an expressive increase of the positivity rate from 8 to 9.5% and from 12.4 to 14.8.9% when one, three, six, and 12 KK slides were examined in individuals from the Buriti Seco and Morro Grande communities from Pedra Preta, a small village located in the rural area of Montes Claros, state of Minas Gerais, Brazil. These findings demonstrate the importance of evaluating a larger number of samples and slides to reduce the number of false-negative results, given that there is consensus on the limitation of the parasitological technique in detecting individuals with low parasitic burden. Nevertheless, this approach is not applicable in the epidemiological inquiry due to the lack of operability in field.

The positivity of qPCR was higher than that of the KK (24 slides, *p* = 0.053) and SG techniques (*p* = 0.79) and like the “reference test,” with no statistical difference (*p* = 0.91). Espírito-Santo et al. (21) also reported a qPCR positivity rate 6.8 times greater than that obtained by the results of the Kato-Katz and Spontaneous Sedimentation (HPJ) techniques combined (0.9%), in a study performed with 572 residents of a low endemicity area. These discrepant positivity rates were also described in other studies using conventional PCR (17) and PCR-ELISA (19, 20), as well as LAMP targeting 121 bp (36).

The sensitivity of the qPCR was high (96.7%) but the specificity was low (87.2%) when the KK (24 slides) results were taken as reference. Only one participant positive with eggs diagnosed by the Kato-Katz technique was not identified by the qPCR assay, which can be explained by the absence of eggs in the sample examined. The qPCR detected 15 positive participants not identified by the Kato-Katz, by examining 24 slides. This discordance was probably due to the limitation of parasitological technique for detecting parasite eggs in stools samples from residents of low endemicity areas, where most of the carriers present low parasite burden (<100 epg). In these cases, the PCR assay detects more cases of infection than the evaluations of many slides by the Kato-Katz technique, suggesting that it can be a useful diagnostic tool. In contrast, the sensitivity rates were lower (81.4 and 82.6%) and the specificity rates were high (90.4 and 93.4%) when the SG or “reference test” were considered as a reference. Thus, the accuracy ranged from 87.8 to 89.8%, with no statistical difference (*p* = 0.59).

In a cross-sectional population-based study, the qPCR targeting 121 bp was compared with POC-CCA^®^, KK (18 slides), Saline Gradient, and Helmintex techniques. The qPCR assay presented sensitivity of 91.4%, specificity of 86.9%, and Kappa index of 0.71, when the results of the three parasitological techniques were considered as a “reference test.” Moreover, the qPCR assay diagnosed 86.9% of the participants with very low parasite burden (<12 epg) while the POC-CCA^®^ diagnosed 50.8% (37). Other studies with qPCR targeting the *Schistosoma* cytochrome oxidase gene (38), internal transcriber-spacer-2 sequence (ITS2) (39), SSU rRNA from *S. mansoni* (34), and 28S ribosomal RNA (40) have showed better performance of the qPCR compared to the parasitological techniques. *Schistosoma* spp. 28S ribosomal RNA can be quantitatively detected in stool, serum, and urine (40) with higher sensitivity than the Kato-Katz technique.

Furthermore, retrotransposon (SjR2), a portion of a mitochondrial gene (nad1) and cell-free parasite DNA (cfDNA) detection by Droplet Digital PCR (ddPCR) has shown to be applicable to the diagnosis of schistosomiasis (41, 42). Also, the authors highlight that the capacity to measure infection intensity have important implications for schistosomiasis control.

It is difficult to obtain a valid comparison between parasitological techniques and PCR assays since they are methodologies with different principles. These discrepant results may be related to irregular distribution of eggs in the feces when the number of eggs per gram of feces (epg) is small (43). Although the Kato-Katz technique is considered the choice test to diagnose schistosomiasis in fecal samples, it is not characteristic of a “reference test.” A study showed that the qPCR targeting *Schistosoma* ITS2 applied in a population from Senegal (n = 197) and Kenia (n = 760), high and low endemicity areas, respectively, presented 13–15% more positivity regarding the KK technique (two slides) of a single stool sample (39). Moreover, the authors reported that the positivity of the qPCR assay was very similar in both areas.

The presumed cure rate of 100% post-treatment was expected. However, we observed that 5.6, 21.6 and 16.7, 37.9% of the individuals from Tabuas were positive for *S. mansoni* eggs in the stool or with qPCR at 90 and 180 days post-treatment. Similar data were found in a study performed in the residents from the Pedra Preta community, in the municipality of Montes Claros, Minas Gerais, Brazil (44). An explanation for these findings might be the possible reinfection by *S. mansoni*. Moreover, one must consider the possibility of therapeutic failure caused by an incomplete cure due the sub-curative effect of praziquantel when used at usual doses (45). In this study, the sequential qPCR assay from praziquantel-treated participants showed a long persistence of *S. mansoni* circulating DNA, with a negative correlation between the microscopic egg counts (epg) using the KK technique (24 slides) and Ct obtained by the qPCR assay. In contrast, a qPCR assay for the quantitative detection of *S*. *mansoni* and *S. haematobium* DNA in stool samples in the Senegal population showed significant correlation between the qPCR Ct values and microscopic eggs counts for both *Schistosoma* species (38). In this case, we believe that the high positivity rate of 79.5% found by the microscopic egg counts performed on duplicate stool samples favored the positive correlation.

There are insufficient data regarding the clearance of *Schistosoma* DNA post-treatment. However, is necessary to consider the possibility of unisexual *Schistosome* infection. In this case, male or female worms will be able to release antigens and DNA that could be detected by immunological and molecular techniques, respectively (46). It is possible that *Schistosoma* DNA could continue to be released from eggs or killed worms that are withheld in tissue granulomes. Thus, circulating DNA from schistosomiasis patients is not entirely cleared and might be detected by qPCR assays. Wichman et al. (23, 47) proposed that circulating free DNA may be detected in more than 1 year since inactive eggs may release DNA very slowly. In some patients with chronic schistosomiasis, presenting a higher number of *Schistosoma* eggs, circulating free DNA may remain for considerably longer (48). Moreover, the authors highlight that the decrease of *Schistosoma* circulating free DNA pre- and post-treatment may be useful for monitoring patients under therapy.

In Estreito de Miralta, the positivity obtained by the KK technique (24 slides) was high than the SG technique (*p* = 0.76) and qPCR assay (*p* = 0.76). In this district the positivity rate increases constantly according to number of slides examined, disagreeing with the behavior shown in Tabuas. In Estreito de Miralta all participants presented low parasite load and possibly this fact influenced the correlation between the number of slides and positivity rates (**Table 5**).

In both districts (Tabuas and Estreito de Miralta), the high positivity rates for schistosomiasis were found in participants aged from 10 to 19 years, followed by participants aged between 20 and 29 (**Figures 3** and **4**). Burlandy-Soares et al. (49) also found high positivity when using the KK technique in these age ranges for the population of Pedro de Toledo, a low endemicity area of the state of São Paulo, Brazil. These findings clearly show the relevance of these age groups for the disease epidemiology.

Despite the low parasite load presented by the infected participants in Estreito de Miralta, the qPCR presented a positivity rate of 18.3%, approximately twice as high as that obtained by the Kato-Katz technique (two slides), as is performed in the Brazilian Schistosomiasis Program Control. Thus, approximately 50% of the individuals infected with *S. mansoni* continue to eliminate eggs and contribute for maintaining the transmission of the disease in the area. This evidence emphasizes the urgent need for a more sensitive diagnostic method for surveilling schistosomiasis cases in low transmission areas (25).

Apparently, the sensitivity rates of the qPCR assay (64.3, 69.2, 57.1%) were impaired and the specificity (92.9, 93.1, 94.4%) were favored when considering the KK (24 slides) and SG techniques or “reference test” as true results. These data indicate the influence of individual parasite burden in the performance of the diagnostic techniques used. Some authors reported that false negative results obtained by PCR may be due to a few factors such as inhibition of the amplification reaction by fecal compounds or DNA degradation during transportation of the sample from the field (19, 43). However, all samples from Estreito de Miralta presented amplification of the human β-actin gene, ensuring that negative results correspond to true negative samples for the *Schistosoma* DNA obtained with the qPCR assay. It is a consensus that the specificity of any diagnostic test benefits when it is applied in individuals from low endemicity areas. Also, it is well established that the sensitivity of a diagnostic test benefits when it is applied in individuals from high endemicity areas.

In contrast to the presumed cure rate observed in the population from Tabuas, the presumed cure rates in Estreito de Miralta were high and more consistent in the follow up at 30, 90, and 180 days post-treatment, with no statistical difference (**Table 8**). Surprisingly, the presumed cure rate in the population of Estreito de Miralta district at 30 days post-treatment measured by parasitological techniques and qPCR was only 93.3%. It is opportune to highlight that none of these participants received treatment during the acute phase of the disease, where the juvenile schistosomes are less susceptible to praziquantel (50). Furthermore, an experimental study using the qPCR assay showed that adult schistosomes with 6 weeks post-infection were susceptible to the praziquantel and those juvenile schistosomes with 4 weeks post-infection were not (51).

In conclusion, the diagnostic techniques presented different performance in the populations from two districts (Tabuas and Estreito de Miralta). In Tabuas, the positivity rate was higher, with the participants presenting low, moderate, and high parasite burdens and a considerable percentage of participants positive with *S. mansoni* eggs evaluated by the qPCR assay after undergoing treatment with the recommended dose of praziquantel. In contrast, all participants in Estreito de Miralta were classified as low parasite burden carriers, with less therapeutic failure during the cure assessment performed in this study. Based in these data, we can to suggest that the transmission force of the parasite in Tabuas is higher than that in Estreito de Miralta.

The qPCR was an acceptable diagnostic tool, with added value to microscopy in low endemicity areas for the diagnosis of intestinal schistosomiasis in fecal samples, which makes it particularly useful in low transmission areas, and consequently, in post-treatment settings. Moreover, the qPCR can be multiplexed for the diagnosis of other intestinal parasites making the assay more useful. On other hand, because of the relative high costs, the qPCR assay is not cost-effective for the routine diagnosis of schistosomiasis in endemic countries. However, the qPCR assay has become less expensive over time, with increase of the number of research centers with qPCR infrastructure. Thus, the qPCR assay can be used in the routine diagnosis of helminth infections in countries of low economic power.

## Data Availability Statement

The raw data supporting the conclusions of this article will be made available by the authors, without undue reservation.

## Author Contributions

LMVS aided with the field work and parasitological techniques, and performed the molecular techniques and analyzed the results. CS assisted with the qPCR assay and analyzed the results. ÁAO aided with the enrollment of the participants, assisted with the parasitological techniques, and assisted with the field work. NFFC assisted with the enrollment of the participants and field work. LG assisted with the qPCR assay and critically reviewed the manuscript for intellectual content. AR supported with the study design and critically reviewed the manuscript for intellectual content. PMZC supported with the study design and critically reviewed the manuscript for intellectual content. EO assisted with the study design, data analysis, drafted the manuscript, and critically reviewed the manuscript for intellectual content. All authors contributed to the article and approved the submitted version.

## Funding

This study was financed in part by the Coordenação de Aperfeiçoamento de Pessoal de Nível Superior - Brasil (CAPES) - Finance Code 001; DECIT/CNPq program 2012 #404405/2012-6; PROEP/Pesquisa Clínica Program MCT-CNPq/FIOCRUZ N^o^ 03/2012. EO is supported by CNPq-Brazil (Conselho Nacional de Desenvolvimento Científico e Tecnológico, (Proc. 301159/2016-5).

## Conflict of Interest

The authors declare that the research was conducted in the absence of any commercial or financial relationships that could be construed as a potential conflict of interest.
